# Sheep Lung Segmental Delivery Strategy Demonstrates Adenovirus Priming of Local Lung Responses to Bacterial LPS and the Role of Elafin as a Response Modulator

**DOI:** 10.1371/journal.pone.0107590

**Published:** 2014-09-12

**Authors:** Thomas I. Brown, David S. Collie, Darren J. Shaw, Nina M. Rzechorzek, Jean-Michel Sallenave

**Affiliations:** 1 University of Edinburgh, Medical School, Edinburgh, Scotland, United Kingdom; 2 The Roslin Institute and R(D)SVS, The University of Edinburgh, Easter Bush Veterinary Centre, Roslin, Midlothian, United Kingdom; Facultad de Medicina, Uruguay

## Abstract

Viral lung infections increase susceptibility to subsequent bacterial infection. We questioned whether local lung administration of recombinant adenoviral vectors in the sheep would alter the susceptibility of the lung to subsequent challenge with bacterial lipopolysaccharide (LPS). We further questioned whether local lung expression of elafin, a locally produced alarm anti-LPS/anti-bacterial molecule, would modulate the challenge response. We established that adenoviral vector treatment primed the lung for an enhanced response to bacterial LPS. Whereas this local effect appeared to be independent of the transgene used (Ad-o-elafin or Ad-GFP), Ad-o-elafin treated sheep demonstrated a more profound lymphopenia in response to local lung administration of LPS. The local influence of elafin in modulating the response to LPS was restricted to maintaining neutrophil myeloperoxidase activity, and levels of alveolar macrophage and neutrophil phagocytosis at higher levels post-LPS. Adenoviral vector-bacterial synergism exists in the ovine lung and elafin expression modulates such synergism both locally and systemically.

## Introduction

Evidence from past flu pandemics indicates that secondary bacterial involvement frequently complicates primary viral lung infections and can extend to fatally compromise lung function [1], [2]. Such viral-bacterial synergy is also observed in animals, particularly in domestic ruminants [3], [4], and in pigs [5], [6] where stress and viral respiratory infections commonly predispose towards secondary bacterial pneumonia. The specific nature of the mechanisms involved in enhancing the pathogenicity of what are often commensal bacteria is slowly being unravelled. Such mechanisms may include virus-induced epithelial damage exposing hidden bacterial binding sites [7], [8], viruses altering alveolar macrophage [9]–[12] or polymorphonuclear [13], [14] cell function, decreasing NK-cell activity [15] and/or increasing the production of either pro- or anti-inflammatory cytokines in a manner inappropriate to the clearance of the secondary bacterial infection.

Whilst lung-directed viral gene therapy offers the potential to treat or ameliorate a host of inflammatory, neoplastic and inherited lung diseases [16]–[19] the presence, nature and extent of any synergism that may exist between viruses and bacteria in the respiratory tract may impact on the safety of viral vectors in such a role. Whilst adenoviral vectors are commonly employed in clinical trials concern does exist that adverse effects may accompany their use [20]–[22]. Such effects may arise from the enhanced susceptibility to subsequent bacteria or LPS exposure [23]. Whilst this important phenomenon has been chiefly studied systemically, our own group has extended such studies to address whether potential exists for additive interactions between Ad-vectors and bacterial products at the local lung level. In this regard whilst we and others have shown no deleterious additive effect of Ad vectors in the context of bacterial and/or bacterial LPS instillation in murine lungs [24]–[27] the possible criticism, that extrapolating from such studies directly to man represents a tenuous assumption, drove us to consider the same issue in a large animal model system of arguably more potential relevance for pre-clinical validation of gene therapy protocols. We chose to evaluate these potential interactions and effects using the sheep as a model system. Our experience is that this system offers the advantage of studying local lung responses in relation to concomitant events elicited elsewhere in the lung and allows for the design of protocols wherein each animal serves as its own control [28]–[30]. In addition, the anatomy, physiology and immunological responsiveness of the respiratory system of sheep is more similar to humans than is that of rodents and the repertoire of Toll-like receptors (TLR) in sheep, which are pivotal in the initiation of the innate immune responses, show greater similarity to humans than do those of rodents [31]. Prior experience within our group has also demonstrated in mice that adenoviral expression of the anti-protease elafin is capable of augmenting lung innate immune responses to subsequent lipopolysaccharide (LPS)-mediated injury [25], [26]. As mice do not normally carry a gene for elafin we considered the natural extension of such studies would be to explore the relevance of such observations in the sheep where elafin, in common with observations in people [32]–[36], behaves like a local acute phase reactant [36]. We and others have shown that this molecule is mainly produced at mucosal sites (including the lung) and have pleiotropic anti-microbial/immune-modulating properties in murine models of lung and intestinal inflammation (see Verrier et al (2012) [37] for a recent review).

We therefore used our previously characterised Ad-o-elafin vector [36] to address whether prior adenoviral mediated expression of ovine elafin would modulate subsequent innate immune responses to LPS.

## Materials and Methods

All procedures were approved by the ethical review process of the University of Edinburgh and complied with the United Kingdom Animals (Scientific Procedures) Act 1986. The Ethical Review Committee of the University of Edinburgh reviewed and approved an application to the Home Office for a licence to carry out this research under the Act.

### Adenoviral Constructs

A recombinant adenovirus encoding for the FLAG epitope tagged ovine elafin, (Trappin Ovine Molecule-1 (TOM-1)) cDNA (Ad-ovine elafin) was constructed as detailed elsewhere [36]. Ad expressing green fluorescent protein (Ad-GFP) was a kind gift from F. Graham and R. Marr, Department of Biology, McMaster University, Hamilton, Ontario, Canada. Both viruses were devoid of detectable LPS contamination (data not shown) and had very similar particles/plaque-forming unit (pfu) ratios (99 and 102, respectively).

### ELISAs

A bovine tumor necrosis factor alpha (TNF-α) Screening Set (Endogen, Pierce Biotechnology, Inc., Rockford, IL, USA), used according to the manufacturer's instructions, established levels of TNF-α in bronchoalveolar lavage fluid (BALF) samples. A commercial ELISA kit for human pre-elafin/SKALP (Hycult Biotechnology b.v., AA Uden, The Netherlands) was used according to the manufacturer's instructions to determine elafin levels in BALF samples.

### Production of Mannheimia haemolytica LPS

A 0.5 ml aliquot of M. haemolytica was inoculated into 50 ml of Nutrient Broth and incubated at 37°C overnight. 30 ml of this culture was then used to inoculate 3 litres of Nutrient Broth and incubated at 37°C for 18 hours in an orbital shaker at 100 rpm. The bacteria were then pelleted at 5000 g for 30 minutes and re-suspended in 50 ml of sterile distilled water. This suspension was then warmed to 68°C and an equal volume of 90% aqueous phenol at 68°C was added. The resultant mixture was maintained at 68°C for 10 minutes after which it was placed on ice to allow phase separation. After centrifugation at 5000 g for 30 minutes at 4°C, the upper aqueous phase was collected and dialysed for 36 hours against several changes of distilled water using Spectra/PorR dialysis tubing (3,500 molecular weight cut-off). The resultant solution was then lyophilized and diluted in sterile water to a final concentration of 150 µg/ml.

### Animals

Sixteen commercially sourced crossbred sheep were used in this study. Animals were treated with anthelminthic prior to entry into the study. Freedom from unrelated pulmonary disease was subsequently confirmed at necropsy.

The sheep were anaesthetised and placed in a whole body plethysmograph as detailed elsewhere [29]. Briefly, each animal received 20 mg/kg body weight thiopentone sodium (Intraval sodium; Merial Animal Health Ltd., Harlow, Essex, UK) as a bolus after which the animals were intubated and maintained in a state of general anaesthesia by the use of 2–3% inhaled halothane in oxygen and nitrous oxide. The sheep were then placed in sternal recumbency in a plexiglass whole body negative pressure ventilator and ventilated via the endotracheal tube which was connected to the anaesthetic circuit via a junction in the wall of the plethysmograph. Extracorporeal pressure was varied via a bellows pump (Cuirass, Cape Road, Warwick, UK) allowing the maintenance of a sinusoidal tidal respiratory pattern. Tidal volume and end-tidal CO_2_ were maintained at 10 ml/kg body weight and 4.5–5.5% respectively (CO_2_ was measured using an Oxicap Monitor Model 4700, Ohmeda, Louisville).

### Bronchoalveolar lavage

A flexible fibre-optic bronchoscope (5.3 mm OD)(Model FG-16X; Pentax U.K. Ltd.) was wedged in selected segmental bronchi. Two 20 ml aliquots of normal saline (0.9% NaCl solution) were used to collect BALF from selected lung segments. BALF samples were passed through sterile gauze into a sterile Falcon tube and immediately placed on ice until subsequent analysis.

BALF was spun at 400 g for seven minutes to separate out the cellular fraction. The resultant pellet was re-suspended in sterile phosphate-buffered saline (PBS) and the total cell number counted before subsequent preparation of cytospins for differential cytology. Supernatants were re-centrifuged at 1,000×g at 4°C for 20 min and stored at −70°C. Cells were counted using a Neubauer haemocytometer and values expressed per millilitre BALF. Cyto-centrifuge slides were prepared and stained using Diff Quick stain for differential counts on 500 cells. Cells were classified as neutrophils, macrophages, eosinophils, lymphocytes or mast cells according to standard morphological criteria.

### Culture of BALF cells

Cells from BALF samples were plated out in 48 well tissue culture plates at 50,000 cells per well in RPMI containing 10% foetal calf serum (FCS), penicillin G (final concentration 100 U/ml), streptomycin sulphate (final concentration 100 µg/ml), L-glutamine (final concentration 2 µM) and amphotericin B (0.5%). After 6 hours non-adherent cells were washed off the wells and the adherent cells cultured for a further 7 days in fresh medium. Supernatants were then collected, spun at 13,000 rpm for 1 minute to remove cellular contamination, and stored at −30°C.

### Optimisation of transfection efficiency in cultured alveolar macrophages

Adenovirus/calcium phosphate co-precipitates were formed by incubating, with gentle intermittent agitation, the specific amount of stock adenovirus solution (calculated to yield the requisite plaque-forming units (pfu)) with 1 ml of the freshly made calcium phosphate solution (2.14 µl 2.5 M CaCl_2_ added per ml EMEM medium and vortexed well) at room temperature for 20 minutes before dilution to 5 ml with sterile PBS.

Cells from BALF samples were cultured in 24 well plates at a seeding density of 250,000 per well in RPMI containing 10% FCS, penicillin G (final concentration 100 U/ml), streptomycin sulphate (final concentration 100 ug/ml), L-glutamine (final concentration 2 uM) and amphotericin B (0.5%). After 6 hours non-adherent cells were washed off the wells and fresh medium added to each well.

Ad-GFP MOI (Multiplicity Of Infection  =  ratio of infectious virus particles to cells) 100 with or without calcium phosphate co-precipitation was applied to the cells for 20 minutes before removal and replacement of medium. 24 hours later the monolayers were photographed and infection efficiency assessed by visualisation of GFP using UV-microscopy.

Additionally, Ad-o-elafin was employed to transfect these cultures in a similar manner at MOIs of 100 and 200 both with and without co-precipitation. In this latter instance culture medium was collected after 4 days and elafin content assessed using Western blot analysis (see below).

### Western blot analysis of cell culture supernatants

25 µl of supernatant along with known amounts of purified ovine elafin protein were reduced with 1% dithiothreitol and loaded on 4–12% gradient polyacrylamide gel using the Invitrogen NuPage gel system as recommended by the manufacturer. After running the gel proteins were transferred to Hybond ECL Nitrocellulose membrane (Amersham Pharmacia). Resultant membranes were blocked in 5% skimmed milk powder in TPBS (PBS and 0.1% Tween 20). Membranes were probed overnight at 4°C with Trab-2O monoclonal anti-elafin antibody (HyCult Biotechnology, Uden, the Netherlands) diluted 1 in 500–1000 in TPBS or anti-FLAG monoclonal antibody diluted 1 in 1000 in TPBS. Membranes were then washed in PBS-T before the secondary antibody was applied (Goat anti mouse IgG conjugated to HRP). This was followed by final washing, addition of Western LightningTM Chemiluminescent Reagent Plus (PerkinElmer Life Sciences, Inc.), and exposure to X-omat radiograph-quality film (Kodak).

### Instillation of Adenovirus/calcium phosphate co-precipitates into the lung

A fibre-optic endoscope (5.3 mm OD) (Model FG-16X; Pentax U.K. Ltd.) was advanced and wedged in selected segmental bronchi. The adenovirus was diluted from stock solution into 5 ml sterile phosphate buffered saline (PBS) in order to achieve the required number of active particles (pfu). This 5 ml volume was then instilled into the segment, through a polyethylene catheter passed via the giving port of the endoscope. In order to direct each instillation towards the lung periphery the catheter (OD 1.6 mm) was advanced to the point of obstruction and its position retracted slightly before performing each instillation. Each instillation was performed in a carefully controlled manner in order to avoid flooding proximal to the tip of the endoscope and to facilitate dispersion of the instillate into the periphery of the subtended segment. Each instillation lasted approximately 30–60 seconds. At the end of the instillation air was allowed to enter the giving port of the endoscope to allow equilibration of pressures in the segment and the endoscope thereafter carefully withdrawn. No reflux of the adenovirus/PBS was observed.

### Preliminary investigation of the use of adenoviral constructs in the sheep lung

Because calcium phosphate has been shown in other systems and mammals (mice, human) to improve Ad infections both in vitro and in vivo [38]–[40] two preliminary studies were conducted to firstly examine the benefits of this method with regards infection efficiency in the sheep (protocol 1) and secondly examine the duration of transgene expression following adenovirus-mediated gene delivery to the lung (protocol 2).

Protocol 1: One animal (MN (male neutered); Bodyweight (BW) 44 kg) was anaesthetised and instilled in one lung segment with 1×10^8^ pfu Ad-GFP and in another lung segment with 1×10^8^ pfu Ad-GFP co-precipitated with calcium phosphate. 48 hours after this infection the sheep was killed and cryosections were prepared from the two instilled segments and also a naive segment to allow visualisation of GFP-positive cells as described below.

48 hours after lung segmental instillation of Ad-GFP the animals were killed and the lungs removed and inflated for 2 hours with 4% paraformaldehyde at room temperature. The lungs were then rinsed twice with sterile PBS and inflated with 30% sucrose overnight at 4°C. Small representative portions of lung were carefully dissected and stored at 4°C in 30% sucrose. Small pieces of these portions were then mounted in optimal cutting temperature medium (OCT) and 10 µm sections cut and mounted on lysine- coated slides. The residual OCT was rinsed off with sterile PBS and then the slides were dipped in absolute alcohol to remove salts. The slides were subsequently dipped into 1 µg/ml DAPI (4',6-diamidino-2-phenylindole) in methanol for 2 seconds and then immediately dipped firstly into PBS and then into absolute ethanol before being air-dried. Cover slips were then affixed with DPX mounting fluid and GFP+ve cells counted by direct visualisation with UV-microscopy.

Protocol 2: Seven sheep (3 MN, 4F; BW 50 kg (median), range 32–58) were anaesthetised and each animal instilled with 1×10^8^ pfu Ad-GFP with calcium phosphate (n = 7). After 3, 7 and 10 days these animals were anaesthetised and bronchoalveolar lavage fluid (BALF) collected from adenovirus instilled segments and also naïve segments in the contra-lateral lung to analyse alveolar macrophages for GFP expression.

### Modulation of the local and systemic responses to bacterial LPS by the segmental administration of recombinant adenovirus

**Figure 1:**
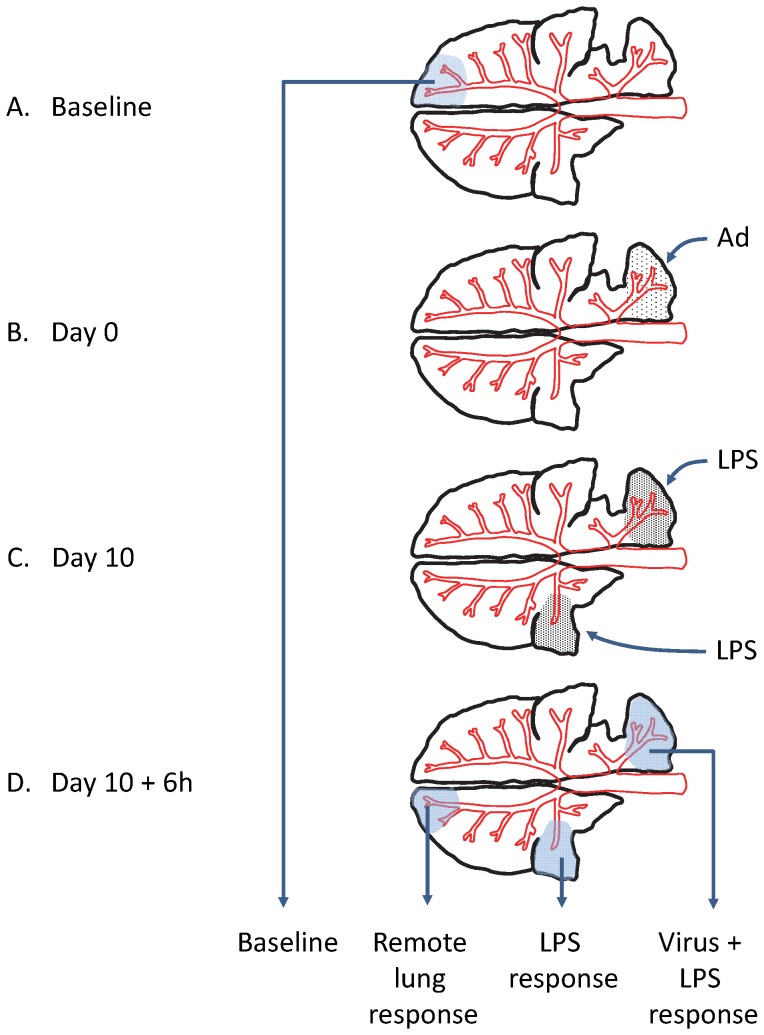
Experimental protocol for the examination of the effect of local up-regulation of ovine elafin on the response to bacterial LPS. A. Baseline sampling of BALF from a lung segment (3–4 weeks before the start of the experiment (day 0) B. Anaesthetised sheep were instilled on day 0 with either Ad-o-elafin-FLAG or Ad-GFP (as a control vector) co-precipitated with calcium phosphate in discrete lung segments. C. Ten days later, bacterial LPS was instilled into the same virus treated segments and also previously naïve segments (Pre-treatment blood samples were obtained immediately prior to anaesthetic induction on Day 10). D. 6 hours after LPS instillation, BALF was collected from these LPS treated segments and also a new ‘remote lung’ segment. The sample categories “Baseline”, “Remote lung response”, “LPS response” and “Virus + LPS response” correspond to the x-axes labels on the boxplots drawn in figure 6. In this design four samples of bronchoalveolar lavage fluid were derived, one sample obtained before any treatment was administered to the lung (Baseline), one sample derived from an area of the lung not subject to any direct treatment (Remote lung response) but obtained after treatments had been administered to other parts of the lung, and further samples from two areas of the lung, the first subject to direct treatment with LPS only (LPS response) and the second subject to treatment with LPS and adenoviral vector (Virus + LPS response).

Three to four weeks prior to study commencement (Fig 1A), BALF was collected from eight female sheep (BW 35 kg (median), range 33–40) to confirm lung health status and provide baseline parameters with respect to the variables under study. Thereafter the sheep entered the experimental protocol as depicted in Fig 1 (B-D). The individual sheep were anaesthetised and each animal instilled with either 1×10^8^ pfu Ad-GFP with calcium phosphate (n = 4) or 1×10^8^ pfu Ad-o-elafin with calcium phosphate (n = 4). Virus was administered into one discrete lung segment of each animal. On day 10 after virus instillation M.haemolytica LPS (3 ml of a sterile solution in water at 150 µg/ml) was instilled into both a virus treated segment and a previously naïve segment in the contra-lateral lung. 6 hours after instillation of LPS the animals were re-anaesthetised and BALF recovered from both the LPS treated segments and also from a spatially distant ‘new’ naïve segment. The BALF samples were assessed for total and differential cell counts, phagocytosis assay, TNF-α and elafin content as detailed elsewhere.

### Blood sampling

Jugular blood was collected by venepuncture into lithium heparin coated vacutainers for the subsequent analysis of total and differential cell counts by a haematology analyser (ABX Pentra 60; Horiba ABX, Montpelier, France).

### Phagocytosis Assays

#### Opsonising E. coli

Four millilitres tetramethylrhodamine conjugated E. coli (E. coli (K-12 strain) BioParticlesR, Molecular Probes, Invitrogen, Paisley, UK) (1×10^7^/ml) incubated with 400 ul heat inactivated serum at 37°C for 45 minutes, were spun at 800G for 15 minutes to form loose pellets. They were washed with RPMI, spun again twice and re-suspended in 4 ml serum-free RPMI to obtain a stock of approximately 10,000/µl which was kept on ice.

#### BALF Phagocytosis assay

50,000 mobile cells were re-suspended into 100 µl RPMI to which 500,000 tetramethylrhodamine conjugated E. coli were added. Identical tubes were incubated on ice and at 37°C. After 20 and 30 minutes each tube was placed on ice and spun at 0°C for 5 minutes at 1300 rpm. Pellets were re-suspended in 500 µl of FACS Lyse solution, vigorously vortexed and incubated on ice for 10 minutes. Following a second spin, pellets were placed on ice and 100 µl trypan blue was added before a further spin and re-suspension in 300 µl RPMI for FACS analysis. Tubes incubated at 0°C served as controls for the test samples incubated at 37°C.

### BALF Myeloperoxidase (MPO) Assay

BALF was spun at 13,000 rpm for 2 minutes and 50 µl added to a 96 well micro-titre assay plate. The MPO activity was determined using a tetramethylbenzidine substrate kit (ImmunoPure, Pierce, Rockford, IL). 100 µl tetramethylbenzidine substrate was added and the plate read by a plate reader at 405 nm with background of 560 nm. The reading was repeated after 10 minutes and the difference between the 2 readings gave a measure of MPO activity. MPO activity was related to the neutrophil count of the BALF sample and the data presented as a percentage change compared to pre-experimental data.

### Statistical Analysis

Three to four weeks after initial baseline (Figure 1A) samples of BALF were obtained; three separate segments of the same lung were exposed to differing combinations of treatment regimes (no treatment, direct exposure to LPS, and direct exposure to virus). At a set interval after these treatments blood and BALF were again collected for analysis (Figure 1D). Blood samples were obtained prior to anaesthetic induction on Day 0 (baseline), Day 10 and on Day 10+6 hours).

#### Systemic Effect

A linear mixed-effect model, where sheep-specific responses were assumed a random effect, was used to analyse the influence of Ad-o-elafin and Ad-GFP and subsequent LPS instillation on blood parameters.

The time point (baseline, Day 10, Day 10+6 hours), treatment (Ad-o-elafin, Ad-GFP) and the interaction between the two were entered as fixed effects of interest. Lymphocyte data required log transformation prior to analysis in order to normalise the residuals. In all cases a P<0.05 was taken to indicate statistical significance.

#### Lung Effect

In addition to the obvious potential association between locally occurring phenomena arising as a direct consequence of locally administered treatments the potential also existed for such treatments to mediate influence on a whole-organ basis and this possibility was addressed through comparison with data obtained at baseline, prior to any treatments being given. As the data relating to the influence of Ad-o-elafin or Ad-GFP expression on LPS-induced local lung inflammation was nested in a hierarchical fashion the analysis was sub-divided into three components (Figure 1):

[i] Samples from un-instilled ‘Remote lung’ segments of the treated lung were compared to samples taken from segments at baseline;
[ii] Samples from un-instilled ‘Remote lung’ segments of the treated lung were compared to samples from lung segments that had been directly exposed to LPS;
[iii] Samples from treated lung segments that had been directly exposed to LPS were compared to samples from segments that had in addition previously been exposed to virus.

A generalised linear mixed-effect (GLME) model where the influence of sheep-specific responses was considered as a random effect was used as segments from the same sheep lung were being compared and the influence of two viruses being considered. For every component of the analysis, virus type, the particular analysis component and the interaction between the two were entered as fixed effects of interest. GLMEs with binomial errors were used for the proportion of AM, PMN and lymphocytes in BALF data, with standard GLMEs used for the other parameters. Total cell counts, AM phagocytosis, BALF Elafin, BALF MPO activity, and BALF TNF-α data required log transformation prior to analysis in order to normalise the residuals. In all cases a P<0.05 was taken to indicate statistical significance.

## Results

### Optimisation of transfection efficiency in cultured alveolar macrophages

**Figure 2:**
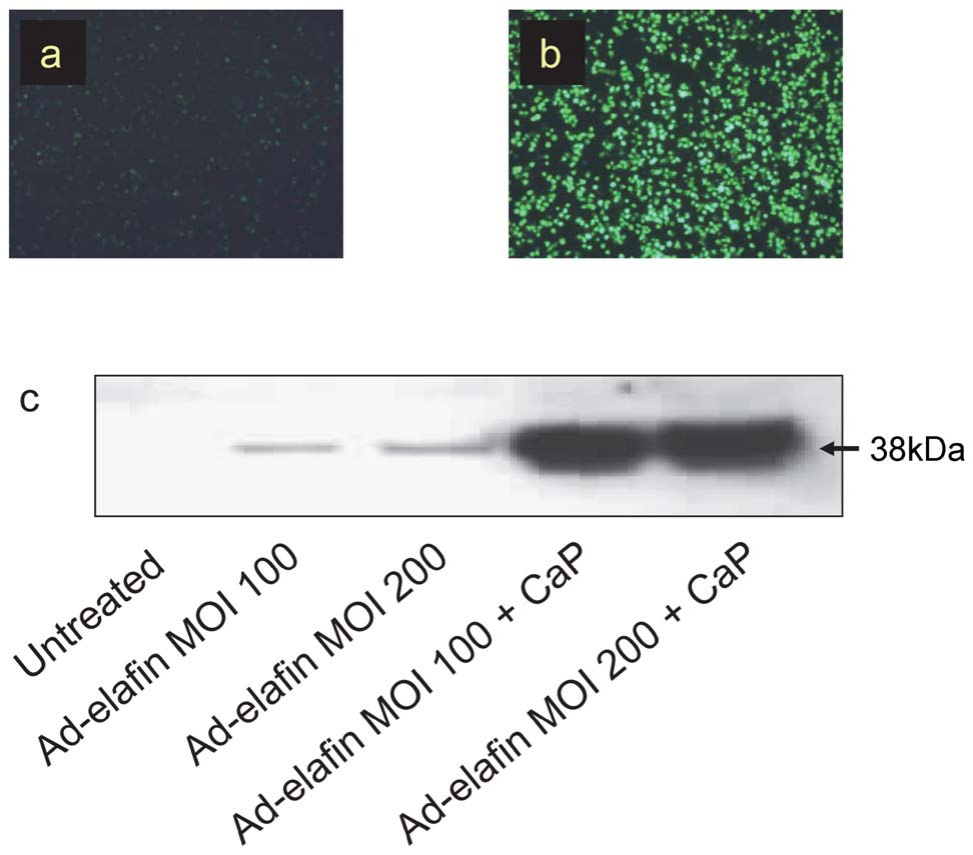
Incorporation of adenovirus into calcium phosphate precipitate increases the infection efficiency of alveolar macrophages in vitro. Ovine alveolar macrophages were cultured at a density of 250,000 per well in 48 well plates and were infected with Ad-GFP and Ad-o-elafin-FLAG at MOI 100 or 200 either pre-complexed with calcium phosphate or as virus alone. (a) – Ovine alveolar macrophages 24 hours after infection with Ad-GFP MOI 100 alone. (b) – Ovine alveolar macrophages 24 hours after infection withAd-GFP MOI 100/calcium phosphate co-precipitate. (c) – Western blot analysis of ovine alveolar macrophage supernatant using Trab-2O antibody 4 days after infection with Ad-o-elafin-FLAG at MOI 100 and 200 either with or without coprecipitation with calcium phosphate. Uninfected alveolar macrophage supernatant is included as a control.

The results of the *in vitro* experiments demonstrated that calcium phosphate up-regulates the infection efficiency of alveolar macrophages'. **Figures 2a and 2b** show the increased infection efficiency of alveolar macrophages by the incorporation of Ad-GFP in a calcium phosphate precipitate. The use of calcium phosphate increased both the number of macrophages infected and the intensity of fluorescence per infected cell. Similarly, **figure 2c** shows that the infection of alveolar macrophages with Ad-o-elafin-FLAG in conjunction with calcium phosphate increases the secretion of ovine elafin.

### Preliminary investigation of the use of adenoviral constructs in the sheep lung

**Figure 3:**
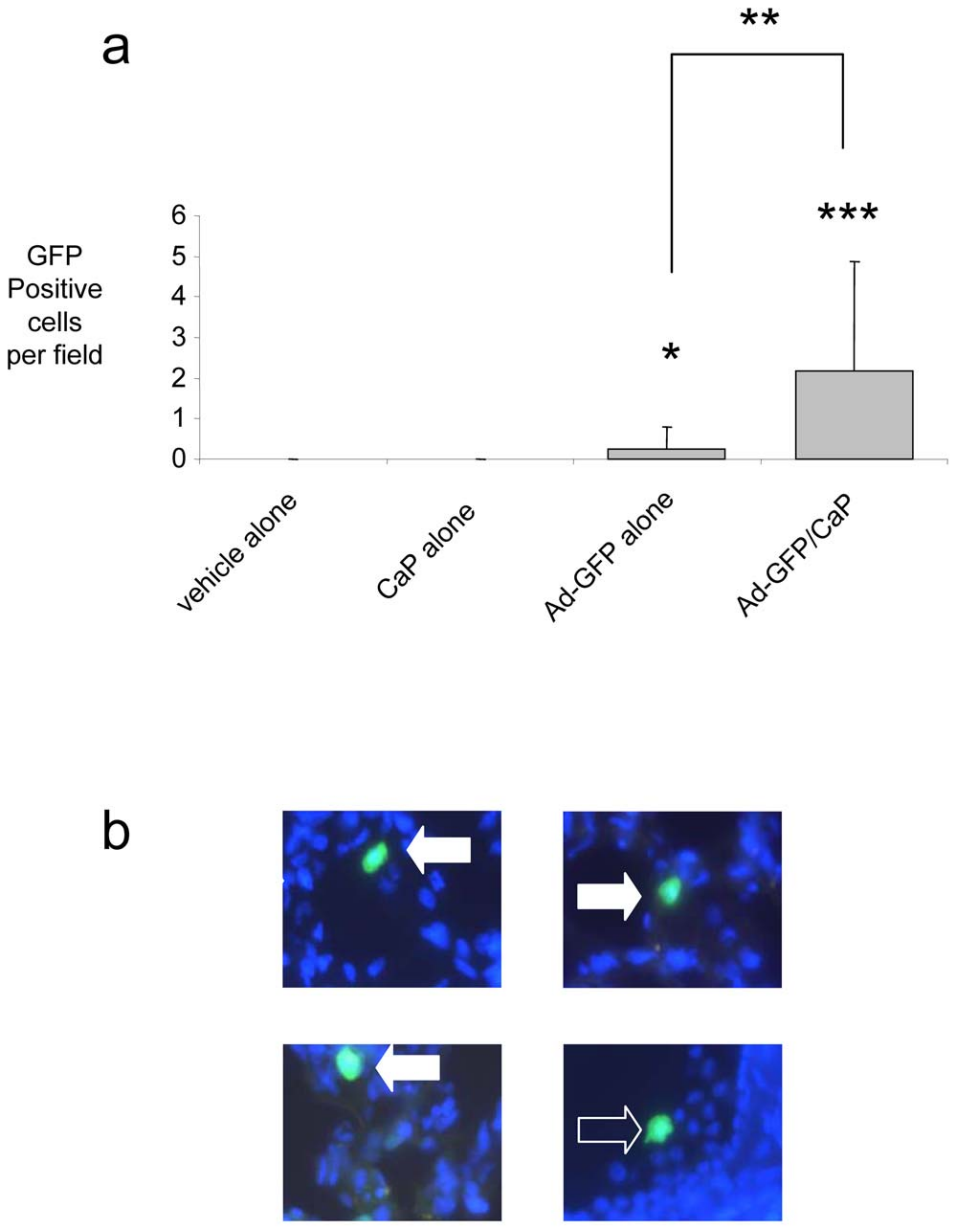
Incorporation of adenovirus into calcium phosphate precipitate increases the infection efficiency of alveolar macrophages and pulmonary respiratory epithelium in vivo. Cryo-sections were prepared from lung tissue 48 hours after instillation of vehicle, vehicle and calcium phosphate, 1×10^8^ pfu Ad-GFP in PBS or 1×10^8^ pfu Ad-GFP after pre-incubation with calcium phosphate. 24 random fields were counted at high power using UV light and the number of GFP+ve cells recorded. (a) – The number of GFP+ve cells is represented here as mean with error bars indicating standard deviation. *, ** and *** indicate P<0.05, P<0.005 and P<0.0005 respectively either comparing values to relevant control (i.e. vehicle alone or vehicle with calcium phosphate) or comparing virus treatment with and without calcium phosphate (indicated by a bar). Significance was calculated by non-paired T Test. (b) – Representative fields from the Ad-GFP/calcium phosphate segment showing infection of Type II epithelial cells (filled arrows) and an alveolar macrophage in an airway (open arrow). Counterstaining of nuclei is with DAPI.

*In vivo* instillation of Ad-GFP incorporated into a calcium phosphate precipitate (see protocol 1, Materials and Methods) led to the increased infection of both alveolar epithelial cells and alveolar macrophages, 48 hrs later, when compared to the instillation of Ad-GFP alone as seen in **figure 3a and 3b**.

**Figure 4:**
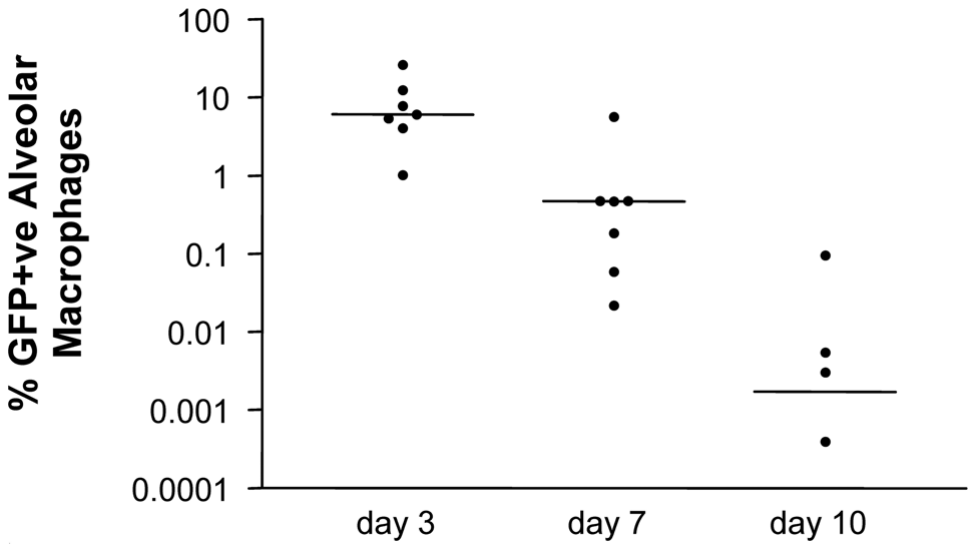
Transgene expression in alveolar macrophages is restricted to the segment infected with adenoviral vector. Alveolar macrophages were collected on day 3, 7 and 10 from sheep infected on day 0 with Ad-GFP/calcium phosphate from both segments infected with adenovirus and from naïve segments and assessed for GFP by UV microscopy after 24 hours in culture. Data is shown as scatter plots with the horizontal bars indicating the median. Macrophages from Ad-GFP infected segments on day 14 were negative for GFP. No GFP+ve macrophages were identified in BALF from any naïve segments at any time point (data not shown). NB Zero values are not shown due to the use of a log scale.

This led us to use this calcium phosphate protocol for the kinetic experiment: GFP positive alveolar macrophages recovered from instilled segments 3, 7 and 10 days after Ad-GFP administration decreased in number with time as shown in **figure 4** (see protocol 2, Materials and methods). At no time point were any GFP+ve cells recovered from any naïve segments. Alveolar macrophages harvested at the same time points and sustained in culture for 7d indicate expression of Ad-o-elafin up to 10 days after instillation (data not shown).

### Modulation of the local and systemic responses to bacterial LPS by the segmental administration of recombinant adenovirus

We, and others, have employed models whereby different treatments are applied to separate lung segments in order to study local lung responses. These models have hitherto proved valuable in the context of defining, at functional, cellular and immune levels, the local lung response to antigen challenge in both experimental animals and in man. Wherein responses can be considered local such an approach allows each animal to serve as its own control, thus reducing the extent of inter-animal variability associated with whole lung studies. Typically such studies have been interpreted through paired (within-subject) or un-paired comparisons between segments. The significant caveat to interpreting such studies arises when the possibility exists that a local treatment could exert an influence across the whole organ, or indeed systemically. In such circumstance, a simple comparison between treated and control segments may fail to demonstrate an effect, not because the treatment has no effect, but because the same influence extends to affect both areas of the lung in the same manner. The experimental design employed in this study (see Fig 1) whereby the viral vector-primed influence on a subsequent local lung response to LPS was the focus, carried this potential, and for this reason we obtained baseline samples and incorporated an overall ‘remote lung response’ effect as the first level of nesting in the analysis. In this way we were able to separate any potential whole-organ effects occurring as a consequence of locally instilled lung treatments from true local effects.

An interval of 10 days elapsed between treatment with the viral vectors and instillation of LPS. Our selection of this interval was influenced by observations in relation to natural and experimentally induced lung disease. Whilst shorter intervals have been employed in sheep to experimentally induce lung disease 5d after exposure to adenovirus [41], other experimental studies in ruminants indicate that viral-bacterial synergism occurs over at least 30-days [42] and naturally occurring outbreaks of pneumonic pasteurellosis in sheep usually occur 10–14 days after a stress [43].

#### Systemic effect

**Figure 5:**
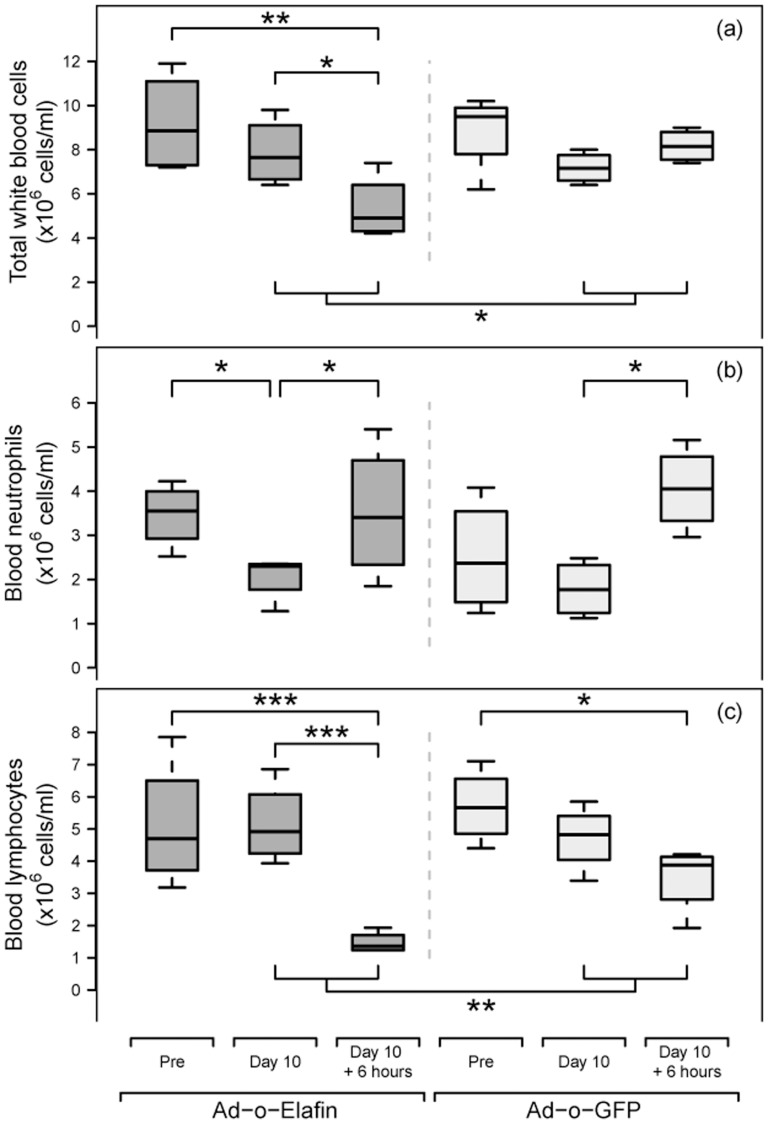
Systemic haematological responses to intrapulmonary adenoviral vector and LPS administration. Heparinised blood was collected by venepuncture at various time points throughout the experimental period (Pre-treatment (immediately prior to collection of baseline BALF samples), d10 and d10+6h). This blood was analysed for total white blood cell numbers, neutrophil numbers and lymphocyte numbers as indicated in a, b and c respectively. The boxplot tails reflect the range of data, the bottom and tops of the boxes the 25th and 75th percentiles respectively, and the thick horizontal line the median values. A linear mixed-effect model, where sheep-specific responses were assumed a random effect, was used to analyse the influence of Ad-o-elafin and Ad-GFP and subsequent LPS instillation on blood parameters. *, ** and *** represent P<0.05 and P<0.01 and P<0.001 respectively.

There were statistically significant differences in total white blood cells between the time points (P = 0.011, **Fig. 5a**). In sheep treated with Ad-o-elafin, relative to baseline total white blood cell counts, statistically significant reductions in total white blood cells occurred six hours after the administration of LPS (Day10 +6 hours, P = 0.004, **Fig. 5a**). Total white blood cell counts at this time point were also significantly lower than counts at the Day10 time point measured just before the addition of LPS (P = 0.027).

There was also a statistically significant interaction between time point and which virus (Ad-GFP or Ad-o-elafin) had been used (P = 0.030), with an observed reduction in total white blood cell counts in response to LPS at Day 10+6 hours compared to Day 10 in Ad-o-elafin treated sheep, but an increase in sheep treated with Ad-GFP (**Fig. 5a**).

There were also statistically significant differences in neutrophils between timepoints (P = 0.008, **Fig. 5b**), with increases in neutrophil numbers in response to LPS (Day 10+6 hours relative to Day 10) apparent for both Ad-o-elafin and Ad-GFP treated sheep (P = 0.042, 0.018, respectively, **Fig. 5b**). Day 10 neutrophil levels in Ad-o-elafin were also significantly lower than pre-treatment levels (P = 0.047, **Fig. 5b**).

Finally, statistically significant differences in lymphocyte numbers between time points occurred (P<0.001, **Fig. 5c**), with pre-treatment levels higher than Day 10+6 hours in both Ad-o-elafin (P<0.001) and Ad-GFP (P = 0.036) treated sheep (**Fig. 5c**). In addition, Day 10 levels in Ad-o-elafin were higher than Day 10+6 hours (P<0.001). There was also a statistically significant interaction between time point and treatment (P = 0.017) with a larger decline in lymphocyte numbers in response to LPS (Day 10+6 hours relative to Day 10) being apparent for sheep treated with Ad-o-elafin than for Ad-GFP (**Fig. 5c**).

#### Lung Effect

**Figure 6:**
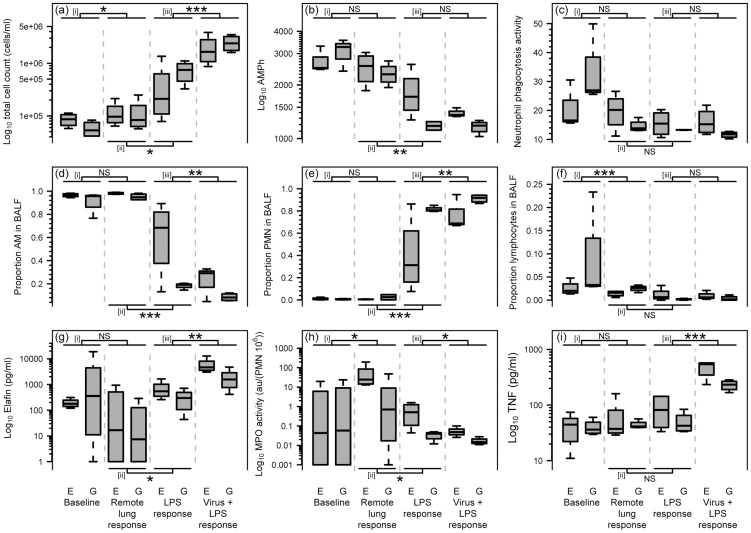
Local lung response to the instillation of adenoviral vectors and/or LPS. Boxplots depicting the (a) log10 Total cell counts (cells/ml); (b) Alveolar macrophage phagocytic activity (AMPh) (geometric mean fluorescence units); (c) neutrophil phagocytic activity activity (geometric mean fluorescence units); (d) proportion of AM in BALF; (e) proportion of PMN in BALF; (f) proportion of lymphocytes in BALF; (g) log10 Elafin (pg/ml); (h) log10 MPO activity (absorbance units (au)/PMN * 10^6^); and (i) log10 TNF-α (pg/ml) data arising from analysis of BALF sampled from differentially treated lung segments of sheep exposed to either Ad-o-elafin (Virus E) or Ad-GFP (Virus G). The boxplot tails reflect the range of data, the bottom and tops of the boxes the 25th and 75th percentiles respectively, and the thick horizontal line the median values. The first pair of boxplots adjacent to the Y-axis reflect baseline data collected before lungs were exposed to treatment (Baseline). The next adjacent pair of boxplots depict data collected from non-treated control lung segments after other segments in the same lung had received different treatment regimes (Remote lung response). The third pair of boxplots reflect data collected from lung segments exposed to LPS six hours previously (LPS response). The last pair of boxplots reflect data collected from segments that had, in addition to LPS treatment six hours previously, been treated with adenoviral vectors ten days before, (Virus + LPS response). The results of statistical comparisons [i]-[iii] between these nested data sets are depicted by the interconnected horizontal lines above and below each separate graph (NS  =  no significant difference, *, ** & *** = P<0.05, 0.01 and 0.001 respectively). The bold font is simply used to emphasise the boxplot pairs across which the above statistical comparisons are directed. Whilst not explicitly annotated in this figure the virus-primed LPS-induced decrease in BALF MPO activity (6h) resulted in levels that were significantly greater for segments exposed to Ad-o-elafin (P = 0.016) when compared to segments treated with Ad-GFP, and levels of AM (6b) and PMN (6c) phagocytosis after LPS were significantly increased in segments exposed to Ad-o-elafin when compared to Ad-GFP (P<0.023).

The potential for the various treatments to have a ‘global’ or ‘whole organ’ effect and thereby influence the variables under study was assessed by comparing data from the control “Remote lung response” segments of the treated lungs to baseline data from separate segments obtained prior to any treatments being administered. A statistically significant increase in total cell counts (P = 0.025; **Fig. 6a** [i]) and MPO activity (P = 0.040; **Fig. 6h** [i]) and decrease in the proportion of BALF lymphocytes (P = 0.026; **Fig. 6f** [i]) occurred in Remote lung response segments relative to baseline. No other statistically significant differences in any other parameter occurred in Remote lung response segments relative to baseline as a consequence of treatment (P>0.070). The nature of the virus used (Ad-o-elafin or Ad-GFP) had no statistically significant effect on any of the parameters, whether in relation to absolute values (P>0.131), or the magnitude of response occurring as a consequence of lung treatment (P>0.069).

Relative to remote lung response segments - exposing segments to LPS resulted in statistically significantly increased total cell counts (P = 0.011; **Fig. 6a** [ii]), BALF elafin (P = 0.042; **Fig. 6g** [ii]), and proportion of PMN (P<0.001; **Fig. 6e** [ii]), but decreased AM phagocytosis (P = 0.002; **Fig. 6b** [ii]), BALF MPO activity (P = 0.037; **Fig. 6h** [ii]) and proportion of AM (P<0.001; **Fig. 6d** [ii]).

Exposing segments to LPS did not significantly influence PMN phagocytosis, the proportion of lymphocytes or amount of TNF-α present in BALF (P>0.070, **Figs. 6c, 6f and 6i** [ii]). The nature of the virus used (Ad-o-elafin or Ad-GFP) had no statistically significant effect on any of the parameters, whether in relation to absolute values (P>0.054), or the magnitude of response occurring as a consequence of LPS treatment (P>0.060).

Exposing segments to virus statistically significantly altered the response of those segments to subsequent administration of LPS. This change took the form of increased total cell counts (P<0.001; **Fig. 6a** [iii]), BALF elafin (P = 0.004; **Fig. 6g** [iii]), BALF TNF-α (P<0.001; **Fig. 6i** [iii]) and proportion of PMN (P = 0.004; **Fig. 6e** [iii]), but decreased BALF MPO activity (P = 0.036; **Fig. 6h** [iii]) and proportion of AM (P = 0.003; **Fig. 6d** [iii]) as a consequence of LPS exposure. Prior exposure of segments to virus did not statistically significantly influence the extent of LPS-induced reduction in AM phagocytosis, nor influenced the observed lack of effect of LPS on PMN phagocytosis or the proportion of lymphocytes in BALF (P>0.116; **Figs. 6b, 6c and 6e** [iii]).

The nature of the virus used did appear to significantly impact on the response to LPS. The aforementioned virus-primed LPS-induced decrease in BALF MPO activity resulted in levels that were significantly greater for segments exposed to Ad-o-elafin (P = 0.016, **Fig. 6h**) when compared to segments treated with Ad-GFP.

In addition, whilst prior exposure of segments to virus did not appear to significantly influence the extent of LPS-induced reduction in AM phagocytosis nor influence the observed lack of effect of LPS on PMN phagocytosis, levels of AM and PMN phagocytosis after LPS were significantly increased in segments exposed to Ad-o-elafin when compared to Ad-GFP (P<0.023; **Figs. 6b, c**).

The nature of the virus used did not significantly impact on the post-LPS levels of any of the other parameters under study (P>0.053), and neither were there any other statistically significant interaction demonstrable between virus type and the magnitude of the LPS-induced response (P>0.100).

## Discussion

Whilst the concept of prior exposure to virus priming the innate immune response to subsequent bacterial products is widely acknowledged [5], [6] this is less well established in the specific case of adenoviruses where the potential mechanisms involved in such priming are largely open to speculation. Regardless of any potential long-term influence, the short term effects of treatment with adenoviral vectors are likely to include the induction of inflammatory and immune responses. As well described [20], [44], [45], innate mechanisms initiate from recognition of pathogen-associated molecular patterns leading to signalling cascades and the expression of pro-inflammatory cytokines, type I interferons and activation of innate and adaptive immunity. Indeed, recent studies have shown that a critical aspect of the murine immune response to adenoviral vectors was the expression of adenoviral type I interferons with mechanisms of in vitro induction mediated by either TLR9 in plasmacytoid dendritic cells (DCs) or cytosolic detection of adenoviral DNA in non-plasmacytoid DCs [44]. Also, TLR-independent mechanisms of adenovirus recognition and type I interferon secretion have been described in splenic myeloid DCs, following in vivo systemic Ad administration [46]. Depending on the cell type, the signalling cascades include the induction of MAPK pathways, NFkB and ICAM-1 gene expression [20], [44], [45], [47] as well as the SAPK/JNK pathway [46]. As many of the intracellular pathways are also used by key cytokines responding as part of the innate immune response the opportunity exists for interplay and interaction within cells responding to LPS and previous adenoviral exposure [48]. Indeed, recently, Fejer et al have shown that systemically- administered Ad vectors lead to LPS hypersensitivity via IFN-γ and epigenetic changes at the TNF-α promoter [46]. Whilst these observations merit further study with respect to identifying whether organ-specific mechanisms operate in murine systems we and others have previously shown that such Ad vector-evoked hypersensitivity may not operate in the context of lung bacterial infection or LPS administration [24]–[26], [49]. To address this issue in an animal model more relevant to pre-clinical studies, we have chosen the sheep, a model amenable to a lung segmental approach to potentially differentiate between local and systemic effects.

Our data indicate that precipitating adenovirus with calcium phosphate is, as in other systems described in the literature, an effective method of optimising transfection efficiency in the sheep, particularly in alveolar macrophages, but also in alveolar epithelial cells (**Figs. 2** **and** **3**). That this optimisation strategy was not associated with any increase in inflammation or cytotoxicity over adenovirus alone was also confirmed (data not shown). We saw no evidence for infection of airway epithelial cells (**Fig. 3b**). This observation contrasts with those of Fasbender et al. [38] who demonstrated that the incorporation of Ad into a calcium phosphate precipitate markedly enhanced the efficiency of gene transfer to airway epithelia both in vitro and in vivo, in mice. Whether this contrast in targeting reflects delivery issues, or species-specific promoter tropisms remains unknown.

Adenoviral dose selection in this study was based on previous unpublished data (Brown, T.I. (2005) Anti-protease gene therapy in the lung. PhD thesis. University of Edinburgh) which indicated that at the dose used in this study (1×10^8^ pfu Ad co-precipitated with calcium phosphate), only very local minor inflammation was elicited (PMNs <9% @ 48h) and was expected to resolve rapidly such that no long-term overt inflammatory sequelae would impact on the subsequent response to LPS in the treated segments ten days later. Clearly however, such a strategy will be insensitive to changes elicited at a molecular level and it would be naïve to assume that lack of influence at a phenotypic level would be a robust indicator of true ‘lack of effect’.

Indeed, we establish here that previous exposure to an adenoviral vector per se potentiates the local lung inflammatory (TCC, PMN%, elafin & TNF-α levels) response to LPS (**Figs. 6a, 6e, 6g and 6i** [iii]). It should be noted, that although, the Trab-2O anti-elafin antibody does not discriminate endogenous from Ad-produced ovine elafin, we previously confirmed that lung Ad- instillation induces very little inflammation *per se* at the dose used in these experiments (data not shown). The increase in o-elafin production between the ‘LPS response’ and the ‘Virus + LPS’ arms of the experiment likely reflects the influence of LPS on the Ad-o-elafin construct and is in keeping with our reported findings (see ref 25 herein) that LPS up-regulates Ad-derived transgenes through up-regulating NF-kB (NF-kB responsive sequences are present in the MCMV promoter).

In addition, the decline in the MPO/PMN ratio occurring as a consequence of LPS treatment was more marked for segments previously treated with virus (**Fig. 6h** [iii]). Whilst seemingly at variance with the potentiated inflammatory response, it is conceivable that reduced MPO levels relative to PMN numbers may indicate the recruitment of immature neutrophil precursors into the lung, functionally altered cells, or the accumulation of degranulated but not yet apoptotic neutrophils.

Paralleling our observation, are the data showing that prior infection with RSV resulted in higher bacterial burden in the lungs of mice exposed a week later to Streptococcus pneumonia [13]. That this occurred in the face of a significantly greater influx of inflammatory cells was potentially explained by the finding that recruited neutrophils were functionally altered in containing less myeloperoxidase [13].

Whilst the nature of the virus (Ad-GFP or Ad-o-elafin) used did not significantly influence the PMN influx as a consequence of LPS treatment it would appear that a trend did exist for a greater influx in Ad-GFP treated sheep (**Fig. 6e** [ii]). Taken together with the previous observation it is conceivable that this again reflects a differential response with respect to neutrophil maturity. In that context, the specific influence of Ad-o-elafin in relation to BALF PMN MPO activity suggests that elafin is helping to maintain enzyme activity in these cells after exposure to LPS (**Fig. 6h** [ii]).

Similarly, elafin also had a protective effect in relation to alveolar macrophage phagocytic capacity that occurred as a consequence of LPS exposure (**Fig. 6b**). The influence of LPS in reducing the phagocytic capacity of alveolar macrophages was not dependent on whether the segment had previously been treated with virus. Whilst at variance with some reports suggesting that LPS either fails to influence macrophage function or improves it [50] our data is in agreement with the broader consensus that sepsis leads to impaired alveolar macrophage function [51].

Lymphopenia (two-fold reduction) and neutrophilia (four-fold increase) are commonly observed 6h after local lung instillation of E.coli LPS in sheep (DDSC data not published). Whilst it was anticipated that the low numbers of sheep involved in this study would preclude demonstration of any significant effect in this regard our data indicate a profound and significant five-fold reduction in circulating lymphocytes in only the elafin-treated animals. Indeed this reduction is the major influence behind the significant reduction in total white blood cell numbers occurring in response to local lung administration of LPS (**Fig. 5**). As blood lymphocyte numbers are maintained by recirculation through secondary lymphoid organs the possibility exists that local lung expression of elafin enhances the sequestration of lymphocytes in response to a local lung inflammation or recruits progenitors from the bone marrow and induces their differentiation and recruitment. These data, obtained in the sheep, potentially share a link with our own data obtained in mice, where we showed that over-expression of Ad-elafin in the lungs of mice had an adjuvant effect in a vaccination protocol [52].

The marginal though significant increase in the total cell number and reduced proportion of lymphocytes in BALF, and the increased MPO activity in remote lung segments relative to baseline (**Figs. 6a, 6f and 6h** [i]) indicate that systemic and/or whole organ effects have arisen as a consequence of the treatment regimes applied elsewhere in the lung. Certainly the aforementioned systemic lymphopenia parallels this observation and hints at the possibility that lymphocytes are being selectively sequestrated elsewhere. Whether, and to what extent, the observed remote lung response reflects systemic, whole organ, and/or spillover effects cannot be definitively established in this analysis. However, if spillover of LPS from treated to remote lung segments did occur then it would be reasonable to expect the nature of the remote lung response to be qualitatively similar to that seen in response to LPS instillation. This is not the case, as LPS instillation caused a significant decrease in BALF MPO activity, whereas the remote response was associated with a significant increase in this variable relative to baseline levels.

Taken together, in this sheep model, our results indicate that infection with an adenoviral vector appears to potentiate the inflammatory response to subsequent challenge with LPS.

Whilst the benefits of such augmentation in relation to handling subsequent bacterial infection are unknown in this model the suspicion is raised that adenoviral infection per se may have a negative influence in this regard through interfering with neutrophil function and phagocytic capacity. In contrast, Ad-o-elafin treated sheep restored neutrophil MPO activity and levels of AM and PMN phagocytosis at higher levels post LPS, compared to Ad-control treated animals. This newly described opsonic activity provides further evidence for the potential utility of elafin both as a local anti-LPS/antibacterial agent at mucosal surfaces and also as an influence in shaping adaptive immunity at a systemic level, by mobilising lymphocytes into the general circulation.

Lastly, our strategy of adopting a lung segmental approach to study design and the careful adoption of rigorous statistical method has demonstrated the feasibility of addressing relatively complex issues using a limited number of experimental animals. Such approaches, which are increasingly represented in the respiratory literature, clearly uphold the principles of reduction and refinement in research involving animals and indicate considerable potential for future strategic application.

## Supporting Information

Figure S1 **ARRIVE (Animal Research: Reporting of In Vivo Experiments) checklist.**
(PDF)
